# Exploring reciprocal within‐person relations between proactive employee green behavior and subjective well‐being: A four‐wave longitudinal study

**DOI:** 10.1111/aphw.70084

**Published:** 2025-11-11

**Authors:** Maie Stein, Clara Kühner, Hannes Zacher

**Affiliations:** ^1^ Wilhelm Wundt Institute of Psychology Leipzig University Leipzig Germany

**Keywords:** employee green behavior, environmental norms, longitudinal study, organizational environmental sustainability, pro‐environmental behavior, well‐being

## Abstract

A promising approach for organizations to advance environmental sustainability and ensure the long‐term well‐being of humanity is to encourage employees to proactively address environmental issues. However, the understanding of how proactive employee green behavior (EGB) is associated with the well‐being of employees engaging in such behavior is currently limited. This is problematic, as the potential co‐benefits of proactive EGB and well‐being may motivate employees to engage in proactive EGB and encourage organizations to promote environmental and well‐being goals simultaneously. Based on data from *n* = 1354 employees in Germany collected across four measurement points separated by 3‐month time lags, we examine how proactive EGB and work‐related subjective well‐being (i.e., job satisfaction and positive and negative affect) are related to one another over time. Results of random intercept cross‐lagged panel models did not provide support for effects of proactive EGB on subsequent well‐being. However, higher positive affect predicted a subsequent increase in proactive EGB. Additionally, we found that the positive within‐person association between job satisfaction and subsequent proactive EGB was stronger among employees who perceived stronger organizational pro‐environmental norms. These findings highlight the importance of promoting subjective well‐being and organizational pro‐environmental norms to support employee initiative that addresses environmental issues.

## INTRODUCTION

Climate change poses various direct and indirect risks to human well‐being, such as heat‐related illness, malnutrition, psychological trauma, depression, and anxiety (Doherty & Clayton, 2011; Inauen et al., 2021; Intergovernmental Panel on Climate Change, 2023; Pearson et al., 2023). As both major beneficiaries of natural resources and significant contributors to environmental degradation (Jayachandran, 2022), business organizations must find ways to advance environmental sustainability. Progress in this area largely depends on the collective decisions and actions of individuals across all organizational levels, including employees, managers and leaders (Ones et al., 2018; Zacher et al., 2023). In addition to avoiding environmentally harmful behaviors and performing prescribed work tasks in environmentally friendly ways, individuals can “make things happen” with regard to organizational environmental sustainability by engaging in proactive employee green behavior (EGB; Zacher et al., 2023). Proactive EGB involves self‐initiated and future‐ and change‐oriented actions to address environmental issues in the organization (Bissing‐Olson et al., 2013). For example, employees could encourage their coworkers to conserve energy, recycle, or commute via public transport, or they could initiate broader environmental policies and programs, such as suggesting environmental campaigns or redesigning work processes to increase resource efficiency and reduce waste and emissions.

Due to the importance of proactive EGB for advancing organizational environmental sustainability, much research has focused on identifying individual and contextual factors that facilitate engagement in such behavior (Ones et al., 2018; Unsworth et al., 2021; Zacher et al., 2023). For example, studies suggest that pro‐environmental attitudes and values, as well as environmentally specific organizational support and leadership, are positively associated with proactive forms of EGB (Katz et al., 2022; Norton, Parker, et al., 2015; Zacher et al., 2023, 2024). Although these studies have advanced the understanding of organizational strategies to promote proactive EGB as a means to contribute to long‐term human well‐being, relatively little attention has been paid to examining how proactive EGB is associated with the well‐being of individual employees engaging in such behavior (Stein, Kühner, & Zacher, 2025; Zacher et al., 2023). This is an important oversight, as understanding how proactive EGB is associated with employee well‐being may provide insights into why employees may—or may not—engage in such behavior. Specifically, (anticipated) well‐being benefits of proactive EGB may motivate employees to engage in these behaviors and lead organizational decision‐makers to promote proactive EGB among their employees.

Broader research in environmental psychology suggests that, despite possible inconvenience, costs, and discomfort, engaging in pro‐environmental behavior is associated with higher levels of well‐being (Krumm, 2024; Zawadzki et al., 2020). This positive association is primarily attributed to the moral nature of pro‐environmental behavior, which leads individuals engaging in such behavior to feel good about themselves (Venhoeven et al., 2016). However, individuals may encounter various challenges and barriers to engaging in pro‐environmental behavior in the work context, such as lack of knowledge, skills, and autonomy (Yuriev et al., 2018). Overcoming these barriers and engaging in proactive EGB may require considerable effort from employees. Additionally, proactive EGB involves initiating changes to the work environment (Bissing‐Olson et al., 2013), which can be perceived as disruptive by others who disagree with these changes. As such, these behaviors may incite resistance or negative reactions from supervisors and colleagues (Zacher et al., 2023), which, in turn, may reduce potential well‐being benefits associated with “doing the right thing.” Therefore, it is important to specifically examine how proactive EGB is related to employee well‐being.

Moreover, employee well‐being may not only constitute an outcome of proactive EGB but also a potential predictor. When employees experience lower levels of well‐being, they may lack the psychological resources necessary to invest effort (e.g., time and energy) and deal with the potential personal risks (e.g., negative reactions from coworkers) associated with proactive EGB (Bindl et al., 2012; Bissing‐Olson et al., 2013). In contrast, higher levels of well‐being may encourage employees to engage in more proactive EGB, as they may have the psychological resources required for the effortful goal‐regulation processes involved in proactive behavior (Cangiano & Parker, 2015; Parker et al., 2010).

From this perspective, proactive EGB and employee well‐being are likely reciprocally related to one another over time. However, most studies examining how pro‐environmental behaviors more broadly (Krumm, 2024; Zawadzki et al., 2020), and proactive EGB specifically (Stein, Kühner, & Zacher, 2025), are associated with well‐being have used cross‐sectional data or incomplete panel designs and, thus, focused on between‐person relations. Consequently, the temporal within‐person dynamics among proactive EGB and employee well‐being are so far not well understood.

**FIGURE 1:**
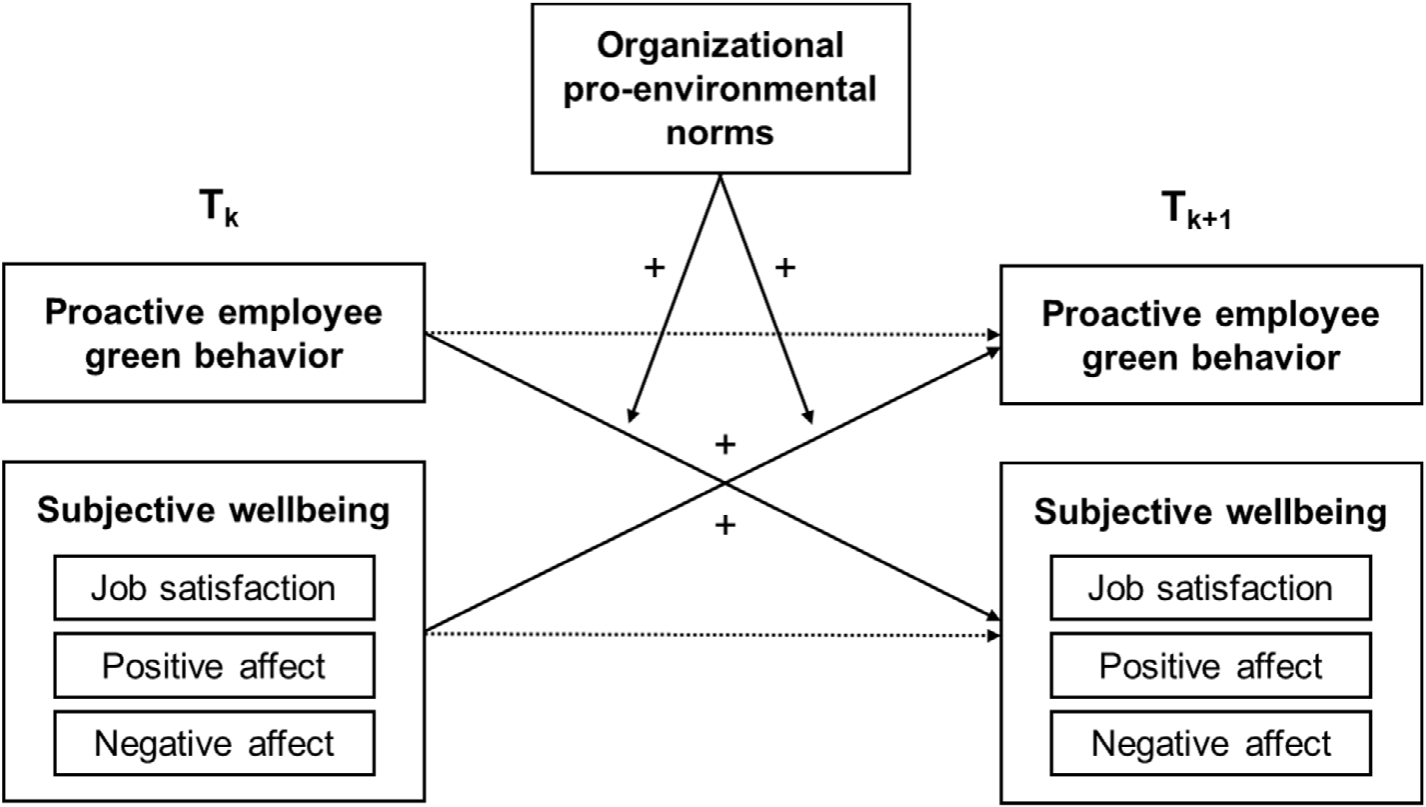
Conceptual model. *Note*: For the sake of clarity and parsimony, only one effect from proactive EGB to subjective well‐being and one effect from subjective well‐being to proactive EGB are depicted. However, the effects for the different subjective well‐being indicators were tested separately.

In this study, we aim to provide insights into how proactive EGB and employee well‐being are dynamically and reciprocally related to one another within individuals over time. We conceptualize employee well‐being as a domain‐specific form of subjective well‐being. Subjective well‐being is an important form of well‐being, as it reflects the key component of hedonic well‐being and has been linked to various health outcomes (Diener et al., 2017). High subjective well‐being is characterized by positive evaluations of one's life and favorable emotional experiences in terms of high positive affect and low negative affect (Diener, 1984). To capture work‐relevant aspects of subjective well‐being, we focus on job satisfaction and positive and negative work‐related affect (Judge & Klinger, 2008). Job satisfaction reflects a domain‐specific form of satisfaction that involves positive affective and cognitive evaluations of one's job (Brief & Weiss, 2002; Locke, 1976). Positive and negative work‐related affect capture the extent to which individuals experience pleasant and unpleasant emotions at work (Brief & Weiss, 2002). By focusing on work‐related subjective well‐being, we aim to capture contextually relevant and temporally dynamic aspects of well‐being that are most likely to influence, and be influenced by, work behavior, such as proactive EGB.^1^ Our conceptual model is shown in Figure 1.

This study contributes to a better theoretical understanding of the intersection between environmental sustainability and well‐being in several ways. By examining within‐person relationships between proactive EGB and subjective well‐being over time, we shed light on how pro‐environmental behavior and well‐being experiences may reinforce one another over time. In doing so, we can capture the dynamic, bidirectional nature of these relationships, which has been largely overlooked in research on pro‐environmental behavior, both in the private domain (Krumm, 2024; Zawadzki et al., 2020) and in the work domain (Stein, Kühner, & Zacher, 2025). This approach also contributes to research on proactivity at work more broadly. Although dynamic feedback loops between proactive work behavior and employee well‐being have been proposed conceptually (Cangiano & Parker, 2015), research has mainly focused on theoretically unidirectional effects of proactive behavior on employee well‐being (e.g., Bohlmann et al., 2021; Cangiano et al., 2019) or vice versa (e.g., Peng et al., 2021). Finally, by examining the moderating role of pro‐environmental organizational norms, we highlight how individual perceptions of the organizational context may impact the relationships between pro‐environmental behavior and well‐being. This contributes to the understanding of organizational factors that could be targeted in interventions aiming to support environmental sustainability and employee well‐being simultaneously.

More broadly, this study aligns with the United Nations Sustainable Development Goals (UN SDGs). Specifically, we address SDG 3 (Good Health and Well‐Being) by examining the role of proactive EGB in influencing subjective well‐being. Additionally, we contribute to several SDGs focused on protecting the natural environment (i.e., SDG 6—Clean Water and Sanitation, SDG 13—Climate Action, SDG 14—Life Below Water, and SDG 15—Life on Land) by exploring ways to promote behaviors that benefit the natural environment.

## THE PRESENT STUDY

In this study, we draw from Cangiano and Parker's (2015) model of proactivity and well‐being as an overarching theoretical framework. Although such effects have rarely been tested empirically, this model suggests that proactive work behavior and employee well‐being are linked to one another via dynamic feedback loops. First, proactive work behavior can increase subjective well‐being by fulfilling basic psychological needs for autonomy, competence, and relatedness (Cangiano & Parker, 2015; Strauss & Parker, 2014).

Proactive EGB is self‐initiated, as employees voluntarily choose to engage in such behavior (Bissing‐Olson et al., 2013). Many individuals perceive pro‐environmental behavior as the morally “right thing to do,” given its benefits for the natural environment and, ultimately, the well‐being of others (van der Werff et al., 2013). As such, employees may perceive their work behavior as self‐directed and aligned with personal values, which can support their sense of autonomy and promote subjective well‐being at work (Deci et al., 2017; Ryan & Deci, 2001). Additionally, proactive EGB is inherently agentic, as it involves challenging the status quo and changing the work environment to make it “greener” (Bissing‐Olson et al., 2013; Stein, Kühner, Katz, & Zacher, 2025). For example, when employees implement improvements to environmental practices, such as reducing waste or promoting environmental sustainability initiatives, they may experience that they are capable of effecting positive change within their work environment. Such experiences of competence have been linked to greater job satisfaction and favorable affective experiences at work (Deci et al., 2017). Proactive EGB also frequently involves social interactions, such as encouraging colleagues to adopt environmentally friendly behaviors or collaborating on environmental initiatives. These social interactions may allow employees to feel connected with others at work, which, in turn, contributes to subjective well‐being by fulfilling the need for relatedness.

Based on these considerations, we hypothesize the following:

Hypothesis 1 At the within‐person level of analysis, proactive EGB is positively related to subsequent subjective well‐being.

Second, Cangiano and Parker's (2015) model also includes a reverse effect from subjective well‐being to proactive behavior. This proposition aligns with broader research on proactivity at work, which suggests that subjective well‐being may serve as a key psychological resource that facilitates proactive behavior by supporting the effortful goal‐regulation processes it requires (Carver & Scheier, 1990; Frese & Zapf, 1994). Accordingly, subjective well‐being may also constitute a predictor of proactive EGB.

Engaging in proactive EGB involves a series of goal‐regulation processes, including identifying environmental problems in the workplace, setting and prioritizing pro‐environmental goals, assessing available resources and potential constraints in the work environment, strategically planning and monitoring the execution of actions, and adjusting behavior based on feedback provided by the work environment (Zacher et al., 2023). For example, an employee might identify excessive paper use as an environmental issue in the workplace and set a goal to address this issue by promoting the adoption of digital document workflows in the organization. Pursuing this goal may require evaluating appropriate digital tools, anticipating potential resistance from colleagues, developing a detailed implementation plan, tracking progress, and modifying the workflows based on feedback.

When employees experience increased levels of subjective well‐being at work, they may be more motivated and better equipped to engage in these goal‐regulation processes. Environmental sustainability represents not only an ethical and legal responsibility for organizations but also a strategic goal (Ambec & Lanoie, 2012). Positive work‐related experiences, such as positive affect and job satisfaction, can lead to increased motivation to contribute to organizational goals by engaging in discretionary work behaviors that extend beyond formal job requirements (Organ & Ryan, 1995; Podsakoff et al., 2000). As such, when employees experience higher subjective well‐being at work, they may be more motivated to engage in proactive EGB, which represents discretionary actions to support organizational environmental sustainability goals.

Additionally, individuals with higher levels of well‐being are more receptive to information from their environment, think more creatively, demonstrate greater optimism regarding their ability to effect change, set more challenging goals and are more persistent in pursuing their goals (Diener et al., 2020; Erez & Isen, 2002; Fredrickson, 2001; Ilies & Judge, 2005). In contrast, impaired well‐being is associated with diminished cognitive functioning (Deligkaris et al., 2014; Shields et al., 2016) and reduced self‐efficacy (Shoji et al., 2016), which may make it more difficult for employees to initiate and sustain the goal‐regulation processes involved in proactive EGB.

Based on these considerations, we hypothesize the following:

Hypothesis 2 At the within‐person level of analysis, subjective well‐being is positively related to subsequent proactive EGB.

Finally, we integrate theorizing on proactive work behavior and employee well‐being (Cangiano & Parker, 2015) with the focus theory of normative conduct (Cialdini et al., 1991) to better understand the contextual factors within organizations that influence the extent to which proactive EGB translates into subjective well‐being and vice versa. Specifically, we position perceived organizational pro‐environmental norms as a potential moderator of the reciprocal relations between proactive EGB and subjective well‐being. Social norms function as implicit rules that define what behaviors are considered appropriate within social contexts, including organizations (Cialdini & Trost, 1998). According to the focus theory of normative conduct, both injunctive norms (i.e., perceptions of behaviors that are widely expected and approved by others) and descriptive norms (i.e., perceptions of behaviors commonly performed by others) shape pro‐environmental behavior by signaling the potential for social (dis)approval of such behavior (Cialdini et al., 1991). In organizational contexts, employees are more likely to engage in proactive EGB when they believe that others in the organization expect and value such behaviors and observe their coworkers engaging in them as well (Norton, Zacher, & Ashkanasy, 2015).

When organizational pro‐environmental norms are strong, it is more likely that employees receive support and recognition from others in the organization (e.g., colleagues and supervisors) for engaging in proactive EGB. Such positive feedback to proactive efforts may further enhance one's feelings of competence and relatedness (Cangiano & Parker, 2015), thereby strengthening the positive impact of proactive EGB on subjective well‐being. Consistent with this notion, cross‐sectional research has found that the positive association between proactive EGB and employee well‐being was stronger among employees who perceived higher levels of organizational support for pro‐environmental efforts (Zhang et al., 2021).

Hypothesis 3 The positive within‐person association between proactive EGB and subsequent subjective well‐being is stronger for employees who perceive stronger (vs. weaker) organizational pro‐environmental norms.

Although subjective well‐being may provide the psychological resources needed to initiate and sustain the goal‐regulation processes involved in proactive EGB, employees may not always be motivated to act on these resources and engage in proactive EGB. This may be because employees can face various individual and contextual barriers to engaging in pro‐environmental behavior in the workplace, such as limited knowledge, lack of skills, or insufficient autonomy and support, which may undermine their perceived control and reduce their likelihood of engaging in proactive EGB (Yuriev et al., 2018).

However, employees may be motivated to overcome these barriers and take advantage of opportunities to engage in proactive EGB when they perceive that organizational norms signal that pro‐environmental behavior is expected and valued within the organization. As such, stronger organizational pro‐environmental norms may increase the likelihood that increased levels of subjective well‐being translate into proactive EGB. This notion aligns with theorizing on proactive work behavior more broadly, which suggests that, in addition to feeling energized to engage in proactive behavior, employees also need to feel capable (“can do” motivation) and perceive a compelling reason to engage in such behavior (“reason to” motivation; Parker et al., 2010). When employees perceive strong organizational pro‐environmental norms, they are more likely to feel capable of engaging in proactive EGB and to perceive such behavior as meaningful.

Hypothesis 4 The positive within‐person association between subjective well‐being and proactive EGB is stronger for employees who perceive stronger (vs. weaker) organizational pro‐environmental norms.

To test our four hypotheses, we adopt a longitudinal design and analytical approach that allows us to examine dynamic within‐person associations between proactive EGB and subjective well‐being over time. Specifically, we use data on proactive EGB and subjective well‐being collected from employees at four measurement points separated by time lags of 3 months. Although there currently is limited theoretical guidance on the timeframes over which reciprocal within‐person effects between proactive EGB and subjective well‐being may unfold, 3‐month time lags are likely appropriate for capturing the dynamic effects among these constructs.

This notion is consistent with proactivity research, which suggests that proactivity is a process (Bindl & Parker, 2011). On the one hand, proactive behavior is inherently future focused, involving anticipatory cognitions and actions intended to bring about change in one's work environment (Grant & Ashford, 2008). As such, the consequences of proactive behavior may not be immediately observable but may emerge over a longer timeframe. On the other hand, the motivational foundations of proactivity, including cognitive and affective goal‐regulation processes (e.g., planning, monitoring, and feedback processing), unfold gradually over time and contribute to the development of personal resources that support sustained engagement in proactive behavior (Bindl & Parker, 2011). Supporting this perspective, longitudinal evidence indicates that affective experiences can predict proactive behavior over periods of 1 to 3 months (Bindl et al., 2012).

The positive effects of proactive EGB on subjective well‐being may take some time to become evident because changes related to environmental sustainability in the work environment, such as the introduction of digital workflows, typically require careful planning and coordination with others in the organization (e.g., supervisors). Additionally, the processes that lead to the satisfaction of basic psychological needs, such as experiencing a sense of accomplishment or positive social interactions with colleagues, are likely to develop over time through repeated engagement in proactive EGB rather than through isolated instances of such behavior across a few days or weeks. Moreover, for subjective well‐being to influence proactive EGB, employees must first identify environmental issues, set pro‐environmental goals related to these issues, and plan actions to address them. Although subjective well‐being can support these processes, they are likely to take some time to unfold and manifest in proactive EGB.

## METHOD

### Transparency and openness

All data, statistical code, and complete results of our analyses are available in the Supporting Information: https://osf.io/f9yw2/. This study was not preregistered due to process constraints (i.e., the data collection was already underway when we developed the idea for this study). Data for this study were collected as part of a larger longitudinal survey study with a total of 10 measurement waves between August 2022 and June 2024. Measurement Waves 1 to 5 were separated by 1‐month intervals, Measurement Waves 5 and 6 were separated by 6 months, and Measurement Waves 6 to 10 were separated by 3‐month intervals. However, our modeling approach required equally spaced measurement points. Additionally, due to practical reasons, the well‐being indicators were not assessed at Measurement Wave 6. Based on our theoretical considerations regarding the timeframes over which the effects between proactive EGB and subjective well‐being may unfold, the current study uses the data collected at Measurement Waves 7 to 10 (i.e., between September 2023 and June 2024). For the sake of clarity, the measurement points considered in the current study are referred to as Time (T) 1 to T4 throughout this article.

Based on the same dataset, eight other studies with completely different research questions than the current study and non‐overlapping substantive variables have been published (Guenthner et al., 2025; Kühner, Rudolph, & Zacher, 2024; Kühner, Stein, & Zacher, 2024; Kühner, Stein, Zacher, & Weiss, 2025; Reindl & Zacher, 2025; Stein, Kühner, Katz, & Zacher, 2025; Stein, Weiss, Kühner, & Zacher, 2025; Zacher & Rudolph, 2023). Another study investigated reciprocal within‐person relations between positive and negative affect and citizenship and counterproductive EGB using monthly data collected at the first five measurement points of the longitudinal study between August and December 2022 (Stein, Kühner, & Zacher, 2025). A data transparency table can be found in the Supporting Information.

### Study design, participants, and procedure

This study was approved by the ethics advisory board of Leipzig University (Protocol ID# 2023.08.01_eb_vv_7 and study title: Environmental Sustainability at Work). Participation was voluntary and informed consent was obtained. We used a four‐wave complete panel design with time lags of 3 months between measurement points. To recruit participants, the professional online panel company Nortstatpanel was commissioned. This online panel company is ISO 20252:2019 certified, which ensures high data quality. To be eligible to participate, participants had to be at least 18 years old and be working at least 20 h per week. The panel company conducted a broad sampling of participants to achieve a diverse representation across various occupations, organizations, and industries in Germany.

The sample considered in this study comprises *n* = 1354 employees who provided complete data on organizational pro‐environmental norms at T1 and at least partial responses to the other substantive variables at T1–T4. Additionally, participants had to correctly answer the two instructed response items (e.g., “Please select ‘strongly agree’”) included in the T1 survey to be included in the final sample; *n* = 272 participants did not answer these items correctly. These items were incorporated based on recommendations to identify careless responders in online panel studies (Meade & Craig, 2012). A total of 86.78% of participants worked in the tertiary sector (i.e., provision of services). Regarding industries, most participants worked in health care and social services (12.85%), followed by manufacturing (11.45%), public administration and defense (10.41%), and other service activities (10.19%). An overview of the demographic characteristics of the sample is shown in Table 1. Additional information can be found in the Supporting Information.

**TABLE 1:** Descriptive statistics for incomplete, complete, and panel responders.

	T1–T4 incomplete (*n* = 513)	T1–T4 complete (*n* = 841)	*p*‐value	T1–T4 panel (*n* = 1354)
Age (years)				
Mean (*SD*)	47.8 (12.0)	49.5 (10.9)	.011	48.9 (11.3)
Median [min, max]	49.0 [18.0, 72.0]	51.0 [19.0, 74.0]		50.0 [18.0, 74.0]
Gender				
Male	237 (46.2%)	416 (49.5%)	.272	653 (48.2%)
Female	275 (53.6%)	424 (50.4%)		699 (51.6%)
Missing	1 (0.2%)	1 (0.1%)		2 (0.1%)
Education				
Lower secondary school	27 (5.3%)	54 (6.4%)	.666	81 (6.0%)
Intermediate secondary school	170 (33.1%)	259 (30.8%)		429 (31.7%)
Upper secondary school	89 (17.3%)	157 (18.7%)		246 (18.2%)
College/university	219 (42.7%)	360 (42.8%)		579 (42.8%)
Missing	8 (1.6%)	11 (1.3%)		19 (1.4%)
Income				
€0–€999	69 (13.5%)	117 (13.9%)	.094	186 (13.7%)
€1000–€1999	116 (22.6%)	188 (22.4%)		304 (22.5%)
€2000–€2999	102 (19.9%)	152 (18.1%)		254 (18.8%)
€3000–€3999	99 (19.3%)	131 (15.6%)		230 (17.0%)
€4000–€4999	38 (7.4%)	95 (11.3%)		133 (9.8%)
€5000–€5999	19 (3.7%)	37 (4.4%)		56 (4.1%)
€6000–€6999	27 (5.3%)	63 (7.5%)		90 (6.6%)
Missing	43 (8.4%)	58 (6.9%)		101 (7.5%)
Industry				
Primary and secondary sector	67 (13.1%)	102 (12.1%)	.672	169 (12.5%)
Tertiary sector	442 (86.2%)	733 (87.2%)		1175 (86.8%)
Missing	4 (0.8%)	6 (0.7%)		10 (0.7%)
T1 proactive EGB				
Mean (*SD*)	2.51 (1.14)	2.57 (1.18)	.314	2.55 (1.17)
Median [min, max]	2.67 [1.00, 5.00]	2.33 [1.00, 5.00]		2.67 [1.00, 5.00]
T1 job satisfaction				
Mean (*SD*)	3.61 (1.08)	3.74 (1.02)	.027	3.69 (1.04)
Median [min, max]	4.00 [1.00, 5.00]	4.00 [1.00, 5.00]		4.00 [1.00, 5.00]
T1 positive affect				
Mean (*SD*)	3.18 (0.937)	3.22 (0.928)	.447	3.21 (0.931)
Median [min, max]	3.20 [1.00, 5.00]	3.20 [1.00, 5.00]		3.20 [1.00, 5.00]
T1 negative affect				
Mean (*SD*)	1.76 (0.777)	1.68 (0.749)	.040	1.71 (0.761)
Median [min, max]	1.40 [1.00, 5.00]	1.40 [1.00, 4.80]		1.40 [1.00, 5.00]
T1 organizational pro‐environmental norms				
Mean (*SD*)	3.11 (0.916)	3.17 (0.940)	.263	3.15 (0.931)
Median [min, max]	3.00 [1.00, 5.00]	3.00 [1.00, 5.00]		3.00 [1.00, 5.00]

*Note*: T1–T4 incomplete = Participants included in the final sample with missing data on the substantive variables at T1–T4. T1–T4 complete = Participants who provided complete data on the substantive variables at T1–T4. T1–T4 panel = Participants included in the final sample. The *p*‐value represents the comparison between incomplete and complete responders using *t* tests for continuous variables and *χ* ^2^ tests for categorical variables.

To examine the patterns of attrition over time, we compared complete panel responders (i.e., participants who provided complete data at T1–T4; *n* = 841) with incomplete panel responders (*n* = 513) in terms of demographics and substantive variables at T1. We conducted *t* tests for continuous variables (i.e., age and T1 substantive variables) and *χ* ^2^ tests for categorical variables (i.e., gender, education, industry, and monthly household income). The results of these comparisons showed that complete responders were slightly older and reported slightly higher job satisfaction and lower negative affect compared to incomplete responders (see Table 1). However, the results of a binary logistic regression model indicated that the demographic characteristics and T1 substantive variables had only limited explanatory power regarding response status (i.e., incomplete vs. complete; Cox–Snell pseudo‐*R* ^2^ = .0143). Thus, we do not consider systematic attrition to be a major concern in our analysis.

### Measures

Reliabilities for the variables measured at T1–T4 are reported as means and ranges of Cronbach's alpha and McDonald's omega values across the four measurement waves.

### Proactive EGB

Proactive EGB was assessed at T1–T4 using three items developed by Bissing‐Olson et al. (2013). The items were preceded by the instruction, “In the past three months ….” The items were “I took a chance to get actively involved in environmental protection at work,” “I took initiative to act in environmentally‐friendly ways at work,” and “I did more for the environment at work than I was expected to.” Responses were scored on a 5‐point scale ranging from 1 (*never*) to 5 (*very often*). Reliability was acceptable (*α*_mean_ = .894, *α*_range_ = .888–.897, *ω*_mean_ = .895, *ω*_range_ = .890–.899).

### Subjective well‐being

Job satisfaction was assessed at T1–T4 using a well‐established single item (Wanous et al., 1997): “All in all, how satisfied were you with your work?” Participants were instructed to think about the past 3 months. Responses were scored on a 5‐point scale ranging from 1 (*very dissatisfied*) to 5 (*very satisfied*).

Positive and negative affect were assessed at T1–T4 with five items each from the short form of the Positive and Negative Affect Schedule (PANAS; Mackinnon et al., 1999; Watson et al., 1988). Participants were asked to indicate how they have felt in the work context over the past 3 months. The items are “inspired,” “alert,” “excited,” “enthusiastic,” and “determined” for positive affect, as well as “afraid,” “upset,” “nervous,” “scared,” and “distressed” for negative affect. Responses were scored on a 5‐point scale ranging from 1 (*not at all*) to 5 (*extremely*). Reliabilities were acceptable for both positive affect (*α*_mean_ = .917, *α*_range_ = .913–.922, *ω*_mean_ = .940, *ω*_range_ = .935–.946) and negative affect (*α*_mean_ = .890, *α*_range_ = .887–.896, *ω*_mean_ = .918, *ω*_range_ = .917–.922).

### Organizational pro‐environmental norms

Organizational pro‐environmental norms were assessed at T1 only using three items adapted from Hoppe et al. (2023). This scale assesses both descriptive norms, reflecting perceptions of typical behavior among employees, and injunctive norms, reflecting perceptions of what employees believe others ought to do regarding environmental protection. The items were preceded by the instruction, “In my opinion, a clear majority of employees in our company/organization ….” The items were “… think it is important to do something to protect the environment,” “… are committed to protecting the environment,” and “… should feel obliged to contribute to environmental protection.” Participants responded on a 5‐point scale ranging from 1 (*strongly disagree*) to 5 (*strongly agree*). Reliability was acceptable (*α* = .829, *ω* = .844).

### Analytical strategy

To test our hypotheses, we used a series of random intercept cross‐lagged panel models (RI‐CLPMs; Hamaker et al., 2015). The RI‐CLPM extends the traditional cross‐lagged panel model by incorporating random intercepts that account for differences at the between‐person level of analysis. By using RI‐CLPMs, we separate out stable between‐person relations from dynamic within‐person sources of variance and account for autoregressive effects. This allows us to obtain estimates of pure within‐person cross‐lagged effects between proactive EGB and the different indicators of subjective well‐being (i.e., job satisfaction and positive and negative affect).

To examine the proposed moderating effects, we followed the two‐step approach outlined by Speyer et al. (2023). First, we computed a baseline model, in which we included the relations between proactive EGB and subjective well‐being, but not the between‐person moderator (i.e., organizational pro‐environmental norms). This model was computed using a maximum‐likelihood estimator with the lavaan package (Rosseel, 2012) in R (R Core Team, 2025) version 4.4.3. Full information maximum likelihood (FIML) was used to account for observed missingness.

Second, we specified a series of six RI‐CLPMs to test the proposed moderating effects of organizational pro‐environmental norms on the within‐person relations between proactive EGB and the three indicators of subjective well‐being. We used separate models for each moderating effect due to the computational intensity of estimating cross‐level moderating effects in RI‐CLPM. As no R packages currently support Bayesian estimation of latent interaction effects, these analyses were conducted in Mplus (Muthén & Muthén, 2017) version 8.11. We used default non‐informative priors and Markov chain Monte Carlo (MCMC) estimation with two chains of 5000 iterations each. Convergence was evaluated based on posterior scale reduction (PSR) values <1.05, and chain mixing was assessed using trace plots. We report unstandardized regression estimates, posterior standard deviations (PSDs), and 95% Bayesian credible intervals for these models.

## RESULTS

Means, standard deviations, and correlations of the substantive variables can be found in Table 2. ICC1 values indicated that a notable amount of variance in the substantive variables resided at the within‐person level of analysis (32.1% for proactive EGB, 40.0% for job satisfaction, 25.6% for positive affect, and 32.4% for negative affect). As an initial step in our analysis, we conducted confirmatory factor analyses (CFAs) to evaluate our measurement model and test longitudinal measurement invariance. The results showed that the proposed five‐factor model including latent factors for proactive EGB, positive affect, negative affect, job satisfaction, and organizational pro‐environmental norms fit the data acceptably (*χ* ^2^(111) = 1382.579, *p* < .001, CFI = .921, TLI = .903, RMSEA = .092, SRMR = .068) and better than an alternative three‐factor model in which positive affect, negative affect, and job satisfaction loaded onto the same latent factor (*χ* ^2^(116) = 5439.6, *p* < .001, CFI = .668, TLI = .611, RMSEA = .184, SRMR = .141, Δ*χ* ^2^ = 4057.0, Δ*df* = 5, *p* < .001). Results of our measurement invariance testing showed that metric invariance was upheld for all multi‐item substantive variables measured across T1–T4. Complete CFA results can be found in the Supporting Information.

**TABLE 2:** Multilevel descriptive statistics for substantive variables and demographic characteristics.

Variable	*M*	*SD*_between_	*SD*_within_	ICC1	1	2	3	4	5	6	7	8	9
1. Proactive EGB	2.546	0.955	0.656	.679		.189*	.323*	.039	.534*	.037	.021	.107*	.056
2. Job satisfaction	3.679	0.801	0.654	.600	.026		.784*	−.644*	.248*	.165*	−.037	.052	.009
3. Positive affect	3.196	0.809	0.475	.744	.049*	.353*		−.438*	.295*	.195*	−.012	.102*	.025
4. Negative affect	1.717	0.615	0.426	.676	.013	−.170*	−.074*		−.054	−.260*	.114*	−.008	.071*
5. Org. pro‐environmental norms	3.150	0.931	‐	‐	‐	‐	‐	‐		.065*	.044	.093*	.076*
6. Age	48.865	11.313	‐	‐	‐	‐	‐	‐	‐		−.041	−.056*	−.159*
7. Gender	0.517	0.500	‐	‐	‐	‐	‐	‐	‐	‐		−.143*	−.121*
8. Income	3.033	1.856	‐	‐	‐	‐	‐	‐	‐	‐	‐		.200*
9. Education	1.991	0.999	‐	‐	‐	‐	‐	‐	‐	‐	‐	‐	

*Note*: Between‐person (within‐person) correlations are above (below) the diagonal. *n*_between_ = 1333, *n*_within_ = 4416. Please note that listwise deletion was used to compute the correlations.

* *p* < .05.

The results of the baseline RI‐CLPM are summarized in Table 3. At the between‐person level, proactive EGB was positively related to job satisfaction (*r*_xy_ = .178, *p* < .001) and positive affect (*r*_xy_ = .324, *p* < .001), but not significantly related to negative affect (*r*_xy_ = .043, *p* = .180). Hypothesis 1 proposed that proactive EGB is positively related to subsequent subjective well‐being. However, the results showed that proactive EGB at *T*_k_ did not significantly predict job satisfaction, positive affect, and negative affect at *T*_k+1_. Thus, Hypothesis 1 was not supported. Hypothesis 2 proposed that subjective well‐being is positively related to subsequent proactive EGB. The results showed that job satisfaction and negative affect at *T*_k_ did not significantly predict proactive EGB at *T*_k+1_. However, we found that positive affect at *T*_k_ positively predicted proactive EGB at *T*_k+1_ (*B* = 0.103, *SE* = 0.039, *p* = .009). Thus, we found partial support for Hypothesis 2.

**TABLE 3:** Summary of focal RI‐CLPM parameters.

Parameters	*B*	*SE*	*z*	*p*	95% CI		*B*_std_
					Lower	Upper	
Covariances between random intercepts							
Proactive EGB–job satisfaction	0.132	0.026	5.165	<.001*	0.082	0.183	0.178
Proactive EGB–positive affect	0.246	0.025	9.775	<.001*	0.197	0.296	0.324
Proactive EGB–negative affect	0.025	0.019	1.341	.180	−0.012	0.062	0.043
Job satisfaction–positive affect	0.484	0.026	18.482	<.001*	0.433	0.535	0.780
Job satisfaction–negative affect	−0.308	0.019	−16.328	<.001*	−0.345	−0.271	−0.647
Positive affect–negative affect	−0.206	0.017	−12.257	<.001*	−0.239	−0.173	−0.423
Autoregressive effects							
Proactive EGB *T*_k_ → proactive EGB *T*_k+1_	−0.015	0.029	−0.522	.602	−0.072	0.042	−0.015
Job satisfaction *T*_k_ → job satisfaction *T*_k+1_	0.094	0.035	2.697	.007*	0.026	0.162	0.099
Positive affect *T*_k_ → positive affect *T*_k+1_	0.116	0.039	2.982	.003*	0.040	0.192	0.119
Negative affect *T*_k_ → negative affect *T*_k+1_	0.002	0.031	0.058	.954	−0.059	0.062	0.002
Hypothesized cross‐lagged effects							
Proactive EGB *T*_k_ → job satisfaction *T*_k+1_	0.002	0.025	0.083	.934	−0.047	0.052	0.002
Proactive EGB *T*_k_ → positive affect *T*_k+1_	−0.019	0.019	−0.990	.322	−0.056	0.018	−0.025
Proactive EGB *T*_k_ → negative affect *T*_k+1_	0.022	0.016	1.361	.173	−0.009	0.053	0.032
Job satisfaction *T*_k_ → proactive EGB *T*_k+1_	0.047	0.028	1.702	.089	−0.007	0.102	0.049
Positive affect *T*_k_ → proactive EGB *T*_k+1_	0.103	0.039	2.620	.009*	0.026	0.179	0.079
Negative affect *T*_k_ → proactive EGB *T*_k+1_	0.059	0.038	1.529	.126	−0.017	0.134	0.041
Other cross‐lagged effects							
Positive affect *T*_k_ → job satisfaction *T*_k+1_	0.158	0.041	3.827	<.001*	0.077	0.238	0.123
Negative affect *T*_k_ → job satisfaction *T*_k+1_	−0.013	0.040	−0.332	.740	−0.092	0.065	−0.009
Job satisfaction *T*_k_ → positive affect *T*_k+1_	0.045	0.022	2.053	.040*	0.002	0.088	0.063
Negative affect *T*_k_ → positive affect *T*_k+1_	−0.056	0.030	−1.854	.064	−0.114	0.003	−0.052
Job satisfaction *T*_k_ → negative affect *T*_k+1_	−0.020	0.018	−1.073	.283	−0.056	0.016	−0.031
Positive affect *T*_k_ → negative affect *T*_k+1_	−0.029	0.026	−1.124	.261	−0.080	0.022	0.033

*Note*: Please note that the standardized effects for the other time lags slightly differ, as the equality constraints applied to the over‐time parameters in the model do not extend to standardized effects. *n* = 1354 employees.

Abbreviations: 95% CI, 95% confidence interval; *B*, unstandardized regression weight; *B*_std_ = standardized regression weight based on T1–T2 effects; *SE* = standard error.

* *p* < .05.

Table 4 contains the relevant parameters of the RI‐CLPMs in which we included the hypothesized moderating effects of organizational pro‐environmental norms. Hypothesis 3 proposed that the positive relation between proactive EGB and subsequent subjective well‐being is stronger for employees who perceive stronger organizational pro‐environmental norms. However, T1 organizational pro‐environmental norms did not significantly moderate the within‐person relations between proactive EGB at *T*_k_ and job satisfaction, positive affect, and negative affect at *T*_k+1_. Thus, Hypothesis 3 was not supported.

**TABLE 4:** Relevant parameters of the six RI‐CLPMs for testing the proposed moderating effects of organizational pro‐environmental norms.

Effect	*B*	PSD	95% CI	
			Lower	Upper
Proactive EGB *T*_k_ → job satisfaction *T*_k+1_	0.004	0.027	−0.048	0.061
Organizational pro‐environmental norms × proactive EGB *T*_k_ → job satisfaction *T*_k+1_	−0.044	0.024	−0.092	0.002
Proactive EGB *T*_k_ → positive affect *T*_k+1_	−0.021	0.020	−0.059	0.020
Organizational pro‐environmental norms × proactive EGB *T*_k_ → positive affect *T*_k+1_	0.032	0.018	−0.003	0.067
Proactive EGB *T*_k_ → negative affect *T*_k+1_	0.020	0.016	−0.012	0.053
Organizational pro‐environmental norms × proactive EGB *T*_k_ → negative affect *T*_k+1_	0.024	0.016	−0.008	0.055
Job satisfaction *T*_k_ → proactive EGB *T*_k+1_	0.048	0.028	−0.006	0.104
Organizational pro‐environmental norms × job satisfaction *T*_k_ → proactive EGB *T*_k+1_	0.077*	0.024	0.028	0.124
Positive affect *T*_k_ → proactive EGB *T*_k+1_	0.116	0.041	0.036	0.198
Organizational pro‐environmental norms × positive affect *T*_k_ → proactive EGB *T*_k+1_	0.047	0.035	−0.022	0.115
Negative affect *T*_k_ → proactive EGB *T*_k+1_	0.061	0.041	−0.018	0.146
Organizational pro‐environmental norms × negative affect *T*_k_ → proactive EGB *T*_k+1_	−0.008	0.038	−0.081	0.069

*Note*: *n* = 1354.

Abbreviations: 95% CI, Bayesian 95% credible interval; *B*, unstandardized regression weight; PSD, posterior standard deviation.

* *p* < .05.

**FIGURE 2:**
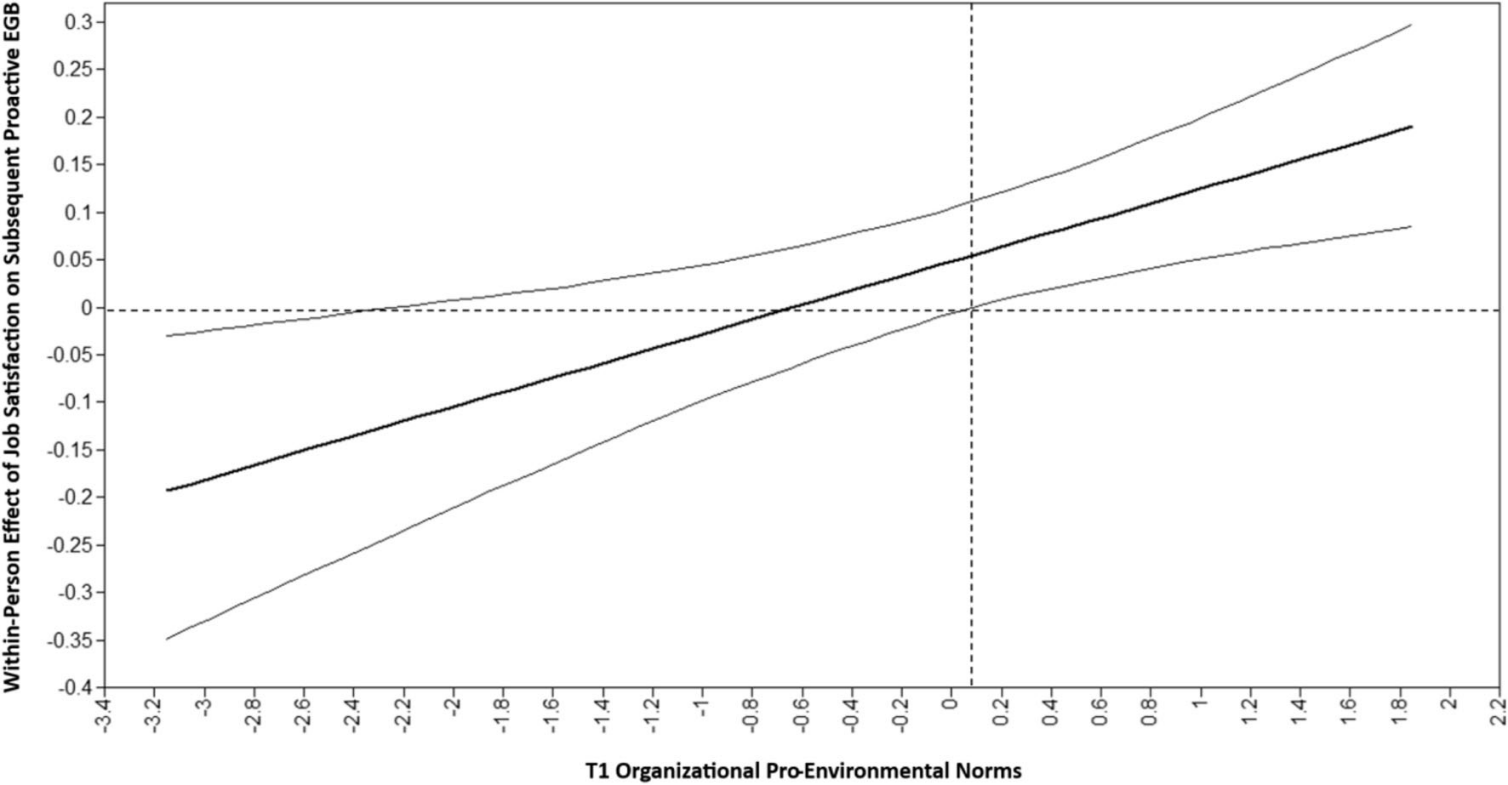
Within‐person relation between job satisfaction and subsequent proactive EGB as moderated by organizational pro‐environmental norms. *Note*: T1 organizational pro‐environmental norms are centered at the mean. Observed values of T1 organizational pro‐environmental norms: min = −2.150, max = 1.850. The thick solid line represents the within‐person effect of job satisfaction on subsequent proactive EGB at different levels of T1 organizational pro‐environmental norms, and the thin solid lines represent the 95% credible bands.

Hypothesis 4 proposed that the positive relation between subjective well‐being and subsequent proactive EGB is stronger for employees who perceive stronger organizational pro‐environmental norms. The results showed that the interaction effect between T1 organizational pro‐environmental norms and job satisfaction at *T*_k_ in predicting proactive EGB at *T*_k+1_ was statistically significant (*B* = 0.077, PSD = 0.024, 95% CI [0.028, 0.124]). Inspection of this interaction effect revealed that the positive relation between job satisfaction at *T*_k_ and proactive EGB at *T*_k+1_ was stronger for employees who perceived stronger organizational pro‐environmental norms (see Figure 2). However, organizational pro‐environmental norms did not significantly moderate the within‐person relations between positive and negative affect at *T*_k_ and proactive EGB at *T*_k+1_. Thus, Hypothesis 4 was only partially supported.

## DISCUSSION

The aim of this study was to provide insights into the relations between proactive EGB and subjective well‐being. Drawing from an integration of theorizing on EGB, proactive work behavior, and social norms, we proposed that proactive EGB and subjective well‐being are positively and reciprocally related to one another over time and that these relations are stronger for employees who perceive stronger organizational pro‐environmental norms.

Contrary to our expectations, our results showed no evidence for effects of proactive EGB on subsequent subjective well‐being. One possible explanation for this finding could be that the effects of proactive EGB on subjective well‐being may manifest over shorter timeframes than the 3 months considered in our study. For example, research suggests that engaging in pro‐environmental behavior can generate positive feelings in the short term (Bissing‐Olson et al., 2016), which may fade away over longer periods. Additionally, individual efforts to change the status quo in organizations may lead to exhaustion and trigger resistance from others in the organization, such as colleagues and supervisors (e.g., Grant et al., 2009; Grant & Ashford, 2008; Reynolds Kueny et al., 2019). Addressing such resistance may require employees to invest additional time and effort and could lead to conflicts with colleagues and supervisors, which can result in diminished well‐being (Harris et al., 2009).

In contrast, the proposed effects of subjective well‐being on proactive EGB were partially supported. Specifically, we found a positive, albeit small (Orth et al., 2024), effect of positive affect on subsequent proactive EGB. This finding replicates earlier research on positive affect and proactive EGB at the day level (Bissing‐Olson et al., 2013) and is consistent with broader research on proactive work behavior (Parker et al., 2010), which suggests that positive affect energizes individuals to recognize opportunities for engaging in proactive behavior, initiate action, and persist in the face of challenges.

Additionally, we found that the positive relation between job satisfaction and subsequent proactive EGB was stronger for employees who perceived stronger organizational pro‐environmental norms. This finding can be interpreted through the lens of social exchange theory (Cropanzano & Mitchell, 2005), which suggests that employees who are satisfied with their jobs may feel an obligation to reciprocate by engaging in behaviors that benefit the organization (Organ & Ryan, 1995). Indeed, this social exchange perspective has also been used in previous research examining relations between job satisfaction and proactive forms of EGB. However, these studies have yielded mixed results (e.g., Kim et al., 2019; Paillé et al., 2016; Paillé & Boiral, 2013). Our findings suggest that these inconsistencies could occur, in part, due to the influence of the broader organizational context. Specifically, when employees perceive strong pro‐environmental norms in their organization, they may view pro‐environmental behaviors as a meaningful way to reciprocate positive work experiences, which, in turn, may increase the likelihood that they engage in proactive EGB.

Interestingly, organizational pro‐environmental norms did not moderate the relations between proactive EGB and subsequent subjective well‐being. One possible explanation for this finding is that such norms can represent a “double‐edged sword” (Bastini et al., 2024; Sparkman et al., 2021). On the one hand, strong organizational pro‐environmental norms may increase the likelihood that employees receive social approval when engaging in proactive EGB, which can increase their sense of competence and relatedness and, thus, their subjective well‐being. On the other hand, strong organizational pro‐environmental norms may lead to perceived pressure to conform to expectations, which may undermine one's sense of autonomy and result in diminished well‐being. Supporting this, research suggests that employees may experience “citizenship fatigue” when they feel pressured to engage in discretionary work behavior (Bolino et al., 2015). Future research should investigate these potential countervailing effects of organizational norms on relations among EGB and well‐being. Future research could also explore additional contextual moderators, such as organizational environmental strategy or supervisor support for environmental sustainability (Ramus & Steger, 2000), which may support engagement in proactive EGB, thereby increasing the extent to which employees derive well‐being benefits from engaging in such behavior.

From a practical perspective, our findings suggest that organizations seeking to advance environmental sustainability should promote employee well‐being. In particular, increasing job satisfaction and positive affect may encourage employees to engage in proactive EGB. To support these experiences among employees, organizations can implement work designs characterized by favorable job characteristics, such as job autonomy, task variety and social support (Parker & Ohly, 2008).

Moreover, our finding that the positive relation between job satisfaction and subsequent proactive EGB was stronger for employees who perceived stronger organizational pro‐environmental norms highlights the importance of developing such social norms and making them salient (see Constantino et al., 2022, for an overview of potential approaches). For example, organizations could showcase positive examples of proactive EGB through internal communication channels, such as newsletters, to reinforce the perception among employees that these behaviors are common and valued within the organization. Additionally, organizations could explicitly communicate expectations regarding EGB, integrate environmental sustainability goals into organizational policies, and recognize and reward employees who engage in environmental initiatives. Importantly, employees themselves can also contribute to shaping organizational pro‐environmental norms by modeling EGB, helping colleagues engage in environmentally friendly behavior, and initiating environmental sustainability initiatives.

In relation to the UN SDGs, our findings underscore the close interconnection between SDG 3 (Good Health and Well‐Being) and the SDGs aimed at protecting the natural environment (i.e., SDG 6—Clean Water and Sanitation, SDG 13—Climate Action, SDG 14—Life Below Water, and SDG 15—Life on Land). The observed positive effects of employee well‐being on proactive EGB suggest that efforts to improve well‐being at work can also advance environmental objectives, reinforcing the need for integrated approaches in line with the UN SDGs.

### Limitations and directions for future research

Despite its strengths, such as the use of a complete panel design with four waves of data collection, this study also has limitations that could be addressed in future research. First, all variables were assessed using self‐report data from employees, which may raise concerns about potential biases, such as social desirability and common method variance. However, the temporal separation of the assessments and decomposition of relations into between‐person and within‐person components help mitigate such concerns (Podsakoff et al., 2012). Additionally, self‐report measures are well‐suited for assessing subjective well‐being due to their internal nature.

Regarding our measure of proactive EGB, one of the challenges is the high level of abstraction in the items, which may lead to varied interpretations depending on the organizational context and the employee's specific job role (Bissing‐Olson et al., 2013). However, similar to measures of general proactive work behavior (e.g., Frese et al., 1997; Griffin et al., 2007; Morrison & Phelps, 1999), the broadness of items helps avoid problems with generalizability across jobs and organizations. Additionally, the relatively low base rate of many specific proactive EGBs (e.g., initiating waste reduction campaigns) complicates measurement, as it limits the ability to capture such behaviors reliably through narrowly defined items. Nevertheless, future research could incorporate more context‐specific, objective measures of proactive EGB (e.g., behavioral tracking of suggestions for improving environmental sustainability in team meetings; Lange, 2025) or use multiple data sources (e.g., colleague or supervisor reports) to provide a more comprehensive assessment of engagement in these behaviors.

Second, we specifically focused on subjective well‐being as a comprehensive indicator of hedonic well‐being (Diener, 1984). However, it is possible that the effects of proactive EGB on eudaimonic forms of well‐being, such as personal growth and purpose in life (Ryan & Deci, 2001), are stronger, given the value‐focused nature of pro‐environmental behaviors. Additionally, our arguments for the positive effects of proactive EGB on subjective well‐being were mainly based on Cangiano and Parker's (2015) model of proactivity and well‐being, which emphasizes the role of basic psychological need satisfaction. However, we did not empirically test these mechanisms, and it is possible that proactive EGB may not always satisfy these needs, such as when others react negatively to such behavior, which could undermine the need for relatedness. Future research should consider both hedonic and eudaimonic well‐being indicators, as well as the role of basic psychological need fulfillment to better understand the potential well‐being benefits of proactive EGB.

Finally, we considered time lags of 3 months between the measurement points to allow for the effects between proactive EGB and subjective well‐being to unfold over time. However, future research could adopt multiple time lags of varying lengths to capture both short‐term and long‐term fluctuations in proactive EGB and subjective well‐being and consider modeling their effects using continuous‐time modeling approaches (Rauvola et al., 2021). Such approaches would advance the understanding of the temporal dynamics of the relations between proactive EGB and employee well‐being, accounting for the possibility that the effects of proactive EGB on well‐being, and vice versa, may unfold over different timeframes.

## CONCLUSION

This study provides insights into the temporal dynamics between proactive EGB and subjective well‐being. Although we found no evidence that proactive EGB increases subsequent subjective well‐being over timeframes of 3 months, our results highlight the important role of positive affect in increasing engagement in proactive EGB and suggest that job satisfaction may increase proactive EGB for employees who perceive strong organizational pro‐environmental norms. Overall, the results emphasize that efforts to improve well‐being at work can also advance environmental objectives, reinforcing the need for integrated approaches in line with the UN SDGs. We encourage future research to include a broader range of well‐being indicators and explore different timeframes to better understand how and when the effects among proactive EGB and employee well‐being manifest.

## CONFLICT OF INTEREST STATEMENT

The authors have no conflicts of interest to disclose.

## ETHICS STATEMENT

This study was approved by the ethics advisory board of Leipzig University (approval number: 2023.0801_eb_vv_7, study title: “Environmental Sustainability at Work”).

## Data Availability

Data, statistical code to reproduce the analyses, and complete results are available in the Supporting Information: https://osf.io/f9yw2/.

## Footnotes

^1^
For the sake of parsimony, we use the term “subjective well‐being” to refer to work‐specific subjective well‐being (i.e., job satisfaction and positive and negative work‐related affect) throughout this article.
